# Integrated vector management for malaria control

**DOI:** 10.1186/1475-2875-7-S1-S4

**Published:** 2008-12-11

**Authors:** John C Beier, Joseph Keating, John I Githure, Michael B Macdonald, Daniel E Impoinvil, Robert J Novak

**Affiliations:** 1 Department of Epidemiology and Public Health, Miller School of Medicine, and the Abess Center for Ecosystem Science and Policy, University of Miami, South Campus, 12500 S.W. 152nd Street, Bldg. A, Miami, FL 33177, USA; 2 Department of International Health and Development, School of Public Health and Tropical Medicine, Tulane University, 1440 Canal Street, Suite 2200 New Orleans, LA 70112, USA; 3 Human Health Division, International Centre of Insect Physiology and Ecology (ICIPE), P.O. Box 30772-00100, Nairobi, Kenya; 4 US Agency for International Development, Global Health Bureau, 1300 Pennsylvania Ave, NW Washington, DC 20523, USA; 5 Department of Veterinary Clinical Sciences, University of Liverpool, 111 Main Building, Leahurst, Neston, Cheshire, CH64 7TE, UK; 6 Department of Medicine, Division of Infectious Diseases and the William C. Gorgas Center for Geographic Medicine, Birmingham, AL 35294, USA

## Abstract

Integrated vector management (IVM) is defined as "a rational decision-making process for the optimal use of resources for vector control" and includes five key elements: 1) evidence-based decision-making, 2) integrated approaches 3), collaboration within the health sector and with other sectors, 4) advocacy, social mobilization, and legislation, and 5) capacity-building. In 2004, the WHO adopted IVM globally for the control of all vector-borne diseases. Important recent progress has been made in developing and promoting IVM for national malaria control programmes in Africa at a time when successful malaria control programmes are scaling-up with insecticide-treated nets (ITN) and/or indoor residual spraying (IRS) coverage. While interventions using only ITNs and/or IRS successfully reduce transmission intensity and the burden of malaria in many situations, it is not clear if these interventions alone will achieve those critical low levels that result in malaria elimination. Despite the successful employment of comprehensive integrated malaria control programmes, further strengthening of vector control components through IVM is relevant, especially during the "end-game" where control is successful and further efforts are required to go from low transmission situations to sustained local and country-wide malaria elimination. To meet this need and to ensure sustainability of control efforts, malaria control programmes should strengthen their capacity to use data for decision-making with respect to evaluation of current vector control programmes, employment of additional vector control tools in conjunction with ITN/IRS tactics, case-detection and treatment strategies, and determine how much and what types of vector control and interdisciplinary input are required to achieve malaria elimination. Similarly, on a global scale, there is a need for continued research to identify and evaluate new tools for vector control that can be integrated with existing biomedical strategies within national malaria control programmes. This review provides an overview of how IVM programmes are being implemented, and provides recommendations for further development of IVM to meet the goals of national malaria control programmes in Africa.

## Background

The control of vector-borne diseases represents one of the greatest global public health challenges of the 21^st ^century. Malaria and other vector-borne diseases contribute substantially to the global burden of diseases and disproportionately affect poor and under-served populations living in tropical and sub-tropical regions [1-5]. In the absence of effective control, these diseases have a major impact on public health and socio-economic development. The standard chemical, biological and physical control measures used to kill mosquitoes and other insects vectors, as well as active and passive case-detection and treatment of human infection, have a long and proven track-record of saving lives. However, the potential benefits of integrating vector control strategies into national health and community systems have not been fully realized, especially in sub-Saharan Africa.

Integrated vector management (IVM) is not a new concept and the basic principles of IVM have been used over the past century in the U.S.A. and elsewhere for mosquito control. An example includes the vast network of Mosquito Abatement Districts throughout the U.S.A. [6-9]. These operations are designed to protect people from nuisance-biting and vector species of mosquitoes and are guided by the following basic principles: 1) to effectively reduce adult vector populations and pathogen transmission; 2) that interventions should be ecologically, environmentally, socially, economically and politically acceptable; 3) that management strategies should not create adverse side effects such as environmental contamination or the development of resistance, nor should they have a negative impact on non-target organisms, including beneficial insects, humans, domestic animals and wildlife; 4) to understand the transmission cycle, the life history of the vector species, and the natural factors regulating vector survivorship are critical, 5) that the most effective programmes develop descriptive and predictive models for population dynamics and transmission potential; 6) to have flexibility in terms of changing strategies and tools in response to surveillance and biological data; and 7) that management strategies should be dynamic and able to respond to the results of an active and sensitive mosquito/pathogen surveillance programme.

All the tactics of standard vector control are included in the IVM approach. IVM goes beyond the integration of traditional control measures to emphasize strategies to make vector control programmes compatible with national health systems. IVM also incorporates decision-making based on human and institutional resources, and engages communities to promote sustainability. IVM encourages integrative, multi-disease approaches and promotes the systematic application of different interventions in combination and in synergy with each other. The framework for implementation strategies extends beyond the health sector and involves multiple stake-holders. To promote success, IVM requires continual monitoring, evaluation and legislation, linked with a strong commitment and concerted action by governments and international organizations [10].

In 2004, the global strategic framework for IVM was prepared establishing new, broad principles and approaches to vector control that are applicable to all vector-borne diseases [10]. IVM was incorporated into a global strategic framework with the underlying rationale that effective control of vector-borne diseases will yield tremendous benefits for health and socio-economic development [11]. As part of the global plan to combat neglected tropical diseases for 2008–2015, the WHO has called for the strengthening of IVM and capacity building as one of the strategic areas for action [12]. In 2008, WHO issued a position statement [13] supporting IVM as set out in the Global Strategic Framework for Integrated Vector Management [10]. This statement is intended to accelerate the development of national IVM policies and strategies, and encourage international organizations, donor agencies and other stakeholders to support capacity building necessary for IVM implementation.

Historically, the implementation of integrated approaches to vector control has been a slow and complicated process. In the 1970's, WHO listed five reasons for the slow uptake of integrated vector control: 1) over-estimation of the requirements of integrated control: "*most programme managers are unaware of this possibility that an integrated approach can, in principle, be introduced without additional resources*"; 2) over-sophistication of integrated control: "*in short, for many mosquito species enough is already known ....to base the introduction of integrated control*"; 3) insufficient conviction of the advantages of integrated control; 4) misconceptions about the use of pesticides in integrated control: *"project managers need to be assured that integrated control does not, nor cannot, mean exclusion of pesticides, but their more rational and correct use."*; and 5) insufficient consciousness of comparative costs, effectiveness, benefits and risks: "*application of pesticides, especially via indoor residual spraying (IRS), frequently becomes a routine practice, regardless of the need or the results obtained*" [14]. Additional reasons for the slow uptake of integrated vector control, as of late, include a lack of capacity building, poorly defined roles for advocacy and legislative activities, and a general lack of intersectoral linkage within the health sector.

Like traditional approaches to vector control, IVM relies on an understanding of how environmental factors affect the distribution and densities of different species of vectors, and how effectively control measures reduce vector-human contact, vector survival and the overall intensity of pathogen transmission. IVM expands on traditional approaches to vector control in that collaboration with local communities, other stakeholders (e.g. ministries of agriculture, environment, or sewer and water-works), and public health regulatory and legislative frameworks support the evolution of vector control. Programmes seeking to implement IVM must appreciate that "*effective IVM requires the establishment of principles, decision-making criteria and procedures, together with time-frames and targets*" [15,16]. These principles should be incorporated into national health policies and supported by legislation and regulation.

**Figure 1:**
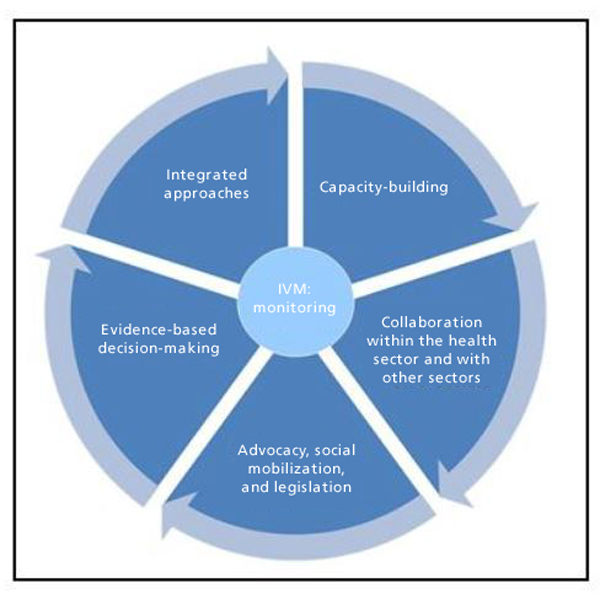
IVM framework and distinguishing characteristics.

Figure 1 illustrates the framework for IVM. Distinguishing characteristics of IVM, as they have been refined over the years, are listed below [10]:

• **Advocacy, social mobilization and legislation**. Promotion and embedding of IVM principles in the development policies of all relevant agencies, organizations and civil society; establishment or strengthening of regulatory and legislative controls for public health and pesticide management; empowerment of communities.

• **Collaboration within the health sector and with other sectors**. Consideration of all options for collaboration within and between public and private sectors; strengthening channels of communication among policymakers, vector-borne disease control programme managers and other IVM partners.

• **Integrated approach**. Ensure rational use of available resources through a multi-disease control approach, integration of non-chemical and chemical vector control methods, and integration with other disease control measures, such as active and passive case detection and treatment.

• **Evidence-based decision-making**. Adaptation of strategies and interventions to local vector ecology, epidemiology and resources, guided by operational research and subject to routine monitoring and evaluation.

• **Capacity-building**. Development of essential physical infrastructure, financial resources and adequate human resources at local and national levels to manage IVM programmes based on needs assessments.

### Why traditional vector control approaches have not solved the problem

Throughout Africa, National Malaria Control Programmes have recently embarked on emphasizing vector control as an essential component [10,17]. Most programmes are using ITNs and/or IRS. When optimally employed, these vector control measures can reduce malaria parasite transmission by 90% or more and can correspondingly reduce malaria incidence, malaria prevalence, high parasite density, and clinical malaria [18-20]. However, questions remain: *Are malaria vector control approaches involving ITNs and IRS sufficient? Where and when are they effective? What else is needed to eliminate the remaining low-levels of transmission? *There is also a strong case for implementing larval control to target immature stages of anopheline mosquitoes to reduce malaria transmission [21-27], but further field trials are needed to show how these additional measures, beyond ITNs and IRS, have a substantial impact on malaria transmission and resulting morbidity and mortality. Clearly, more comprehensive strategies and tools are required when successful malaria control programmes reach a stage when they have to adjust their objectives to strategies for country-wide malaria elimination [28]. Even with substantial success in vector control and renewed enthusiasm, many experts believe that the global eradication of *Plasmodium falciparum *and *Plasmodium vivax *malaria will take at least another 50 years [29].

In developing countries, Ministries of Health are normally charged with mosquito control as part of their overall public health programme, although lines of responsibility and organizational networks vary according to different countries. National guidelines are often developed with input from international organizations, such as the WHO and the CDC; these guidelines influence the strategies and tools employed for control programmes that are carried out by decentralized units of the government. However, there are many reasons why vector-borne diseases remain a major public health problem: 1) entomologists and vector-control specialists often fail to convince decision-makers of the importance of sound vector control; 2) intersectoral collaboration has been recommended as a method to improve mosquito control, but in practice, this type of collaboration has proved difficult; 3) modeling and adaptive management have not been used effectively to quantify the effect of disease and vector control interventions on the economic and social development of communities and nations; 4) failed management of insecticides has created a conflict of "to spray or not to spray"; 5) there has been a failure to connect the true disease burden with other societal burdens to establish the public health importance of vector-borne diseases in low-transmission settings; 6) the interaction between proper utilization of law enforcement and health education has not been explored in sufficient detail; 7) economics plays a central role in mosquito-borne disease control but has not been fully integrated in the evaluation of disease control programmes; 8) entomologists and public health specialists have failed to convince decision-makers that they have an ethical duty to control mosquitoes and the pathogens they transmit; and 9) biomedical strategies, such as active and passive case-detection and treatment, are often used instead of preventative vector control measures, as a result of international donor requirements.

A comparative evaluation of vector-control programmes in five countries [30] suggests that one of the key challenges to successful vector control is the lack of intersectoral collaboration. Many control programmes are structured under Ministries of Health, without organizational links to other government ministries (e.g. environment, education, agriculture and tourism), municipal entities (e.g. engineering, sanitation and water resources) or links to stake-holders in communities (e.g. businesses, educators, community groups and NGO's). Various techniques are being used to control mosquitoes, but the programmatic approaches are generally not linked with mosquito surveillance, fundamental information about the ecology and behaviour of the vector species, or heath-systems data on disease. In addition, a common problem is the lack of stable funding for mosquito control operations and the lack of initiatives to apply leverage in support of mosquito control by other government and community entities. In many respects, the IVM global strategic framework is designed to overcome some of these problems, but is proving difficult in reality.

Progress in implementing IVM continues to be slow. In an Institute of Medicine review of Vector Borne Diseases, David Morens cites five fundamental deficiencies that inhibit our ability to find realistic solutions for vector-borne disease problems: deterioration of public health infrastructure, lack of adequate funding, lack of adequate training and training models, over-specialization in the biomedical sciences, driven by emerging technology and emphasis on the basic sciences, and bureaucratization [31]. If IVM is to be successfully integrated into national malaria control programmes, these deficiencies must be addressed.

### Implementing the IVM approach

A recent report by Chanda et al. [32] describes a comprehensive and highly successful IVM program that has been implemented by the Zambian National Malaria Control Program. Over a relatively short time period, this program has expanded coverage of vector control interventions and leveraged additional resources to build national capacity to the point where they have successfully reduced malaria-related morbidity and mortality. In many respects, the successful implementation of IVM and integrated malaria control in Zambia serves as a prominent success story for all of Africa.

While much remains unknown about the impact of fully developed IVM programmes on malaria transmission [33-36], the IVM approach continues to be a strategy with great promise for disease control in Africa [37-39]. Historically, programmes with IVM elements have brought about significant reductions in vector populations and malaria transmission across a range of transmission settings [40-42]. There is evidence that IVM can complement other existing malaria control strategies (ITN use, access to effective treatment) by avoiding reliance on any single intervention to reduce the burden of malaria [43-45].

Castro and colleagues [46] described a successful intervention in Dar es Salaam, Tanzania, from the late 1980's through the 1990's, with elements of IVM included in the strategy. Gilroy and colleagues [47] reported decreases in malaria incidence and sporozoite prevalence rates in Nigeria using a variety of environmental management techniques, including source reduction and drainage. Utzinger and colleagues [39] reviewed environmental management (EM) activities in peri-urban habitats, towns and areas of disturbed land, around Zambian Copper mines; significant reductions in malaria transmission were achieved over a three to five-year period, using a combination of drainage, filling larval habitats, and bed nets. Keiser and colleagues [37] conducted a meta-analysis on EM studies globally and concluded that EM can have a significant impact on clinical malaria, if EM is appropriate to the eco-epidemiological setting. EM was also used with available larvicides to control malaria in the coastal city of Mombasa [42]. Schliessmann and colleagues [48] used a combination of water drainage techniques and larviciding to reduce the number of malaria cases by 98% from 1969 to 1970 in a coastal flood plain of Haiti. In India, Sharma and colleagues [49,50] reported over 95% reduction in malaria incidence over a four-year period for communities receiving a combination of water-source reduction activities and biological control in larval habitats. Collectively, these studies suggest that multiple vector control strategies may be beneficial when used in combination. Further evaluations are needed to examine the effectiveness of IVM for reducing malaria in Africa. The absence of IVM evaluations, as well as evaluations of packages of interventions, has been noted recently by several researchers [51-53].

One of the largest integrated disease control schemes developed in the 1980's was the Blue Nile Health Project [54]. The 10-year project resulted in the development of better strategies for controlling schistosomiasis, malaria and diarrhoeal diseases in a major area of Sudan.

Sri Lanka has long linked vector control with agricultural development. In the 1970s and 1980s, the Mahaweli River hydroelectric and irrigation development project caused new foci of malaria transmission to emerge around Kandi. The hydrological changes and the relocation of people between malaria endemic and non-endemic areas as a result of resettlement due to the dam reservoirs, contributed to an upsurge in malaria [55] and Japanese encephalitis [56]. This led to a series of integrated vector control strategies based on community participation, ITNs, and larval source management [57]. The work in Mahaweli evolved in 2002 when FAO provided a grant for the initiation of a project on "Integrated Vector and Pest Management" [58]. An important innovation involved participatory education known as the "Farmer Field Schools", making the connections between health, vector-borne disease control and agricultural productivity [59].

Eritrea uses national-level ITN distribution strategies, larval habitat management in malarious areas, and indoor residual spraying in areas with the appropriate eco-epidemiological characteristics. Although Eritrea has seen a marked drop in malaria parasite prevalence and disease, no rigorous evaluation of the IVM activities linked to health outcomes has been done. Among the reasons why impact evaluation exercises have not been done are cost and time. In many instances, not enough resources are allocated for the proper evaluation of an intervention programme, forcing the use of a less rigorous study designs for establishing causal inference.

IVM should be seen as a framework and a strategy rather than a circumscribed, tactical intervention. As such there are a large number of African national malaria control programmes that incorporate some, but possibly not at the present time, all of the IVM elements. "Community-based" IVM programmes incorporating larval source management have recently been implemented in two different settings: in Rusinga Island, Western Kenya [27], and in Dar es Salaam, Tanzania [46,60]. Recent reviews of environmental management [37,39] and larval control [26] contain additional examples. Table 1 provides a list of elements that should be considered when developing IVM approaches. These elements have been used successfully in mosquito-abatement districts in the U.S.A. Most government-operated vector-control programmes in Africa have not implemented the mentioned elements in their entirety or have implemented them with limited efficiency needed for vector and disease management. These elements must be part and parcel of an IVM approach for it to succeed [8].

**Table 1:** IVM checklist for a vector control programme (modified from Hatch 1973 [9] and Challet 1991 [6]).

**Vector control elements**	**General description**	**Specific activities**
Programme administration	• How to manage of vector control?	1. Goal setting
		2. Policy development
		3. Developing staff duties
		4. Risk management
		5. Legislation development and enforcement

Financial and economic assessment	• What is the economic burden of disease and how do you finance vector control?	1. Conduct cost-effectiveness analysis
		2. Financial planning: identifying source of revenue (i.e. taxes, lottery, income generation activities, etc.)

Facilities and equipment	• What elements are needed to do vector control?	1. Selecting and assigning facilities
		2. Determining available and needed equipment

Vector surveillance	• How to measure program effectiveness?	1. Vector population surveillance

Disease detection	• How to determine the quality and quantity of control efforts?	1. Disease detection programme to monitor vector-borne disease parameters

Control activities	• How to establish a guide to control operations that will use the most effective, yet environmentally sensitive method of vector control?	1. Determine appropriate control methods: i) environmental management, ii) biological control, iii) chemical control, iv) legislation
		2. Integrate efforts where possible to achieve synergy

Public education and relations	• How to communicate and interact with community regarding vector control?	1. Personal public education
		2. Printed public education
		3. Customer service
		4. Multimedia education (i.e. TV and internet)
		5. Community outreach

Record/reporting/evaluation	• How to evaluate the programme and achievement of goals?	1. Keeping, compiling and reporting activities
		2. Summarizing annual reports and linking to goals and objectives
		3. Analyze data to evaluate effectiveness
		4. Model data for ecological, human, vector, disease and control trends

Intergovernmental coordination/environmental planning	• How to coordinate activities between stakeholders, which have mutual concerns for vector control, through interagency partnerships?	1. Contact between local and national governments
		2. Planning between vector control staff and environmental development staff
		3. Assessment of environmental impact related to vectors and pest
		4. Develop relationship with conservationist and wildlife enthusiast

Research	• How to build research to determine how local conditions are changing in response to vector control and develop new approaches for control?	1. Incorporate applied basic research in the programme
		2. Review research design and statistical methods

Emergency preparedness	• How to plan on how to dealing with disease situations and natural disasters?	1. Identify responsible agency that coordinates and communicates with the public
		2. Develop vector and disease surveillance that provide early alert to potential emergency conditions
		3. Key control actions to specific situation
		4. Reserve funding for emergency situations

Training and continuing Education	• How to mandate elements for certified personnel?	1. Mandatory training to certify technicians each year
		2. Establish training programmes with sufficient budget
		3. Attend professional and society meetings

The implementation of IVM requires a needs assessment of vector control capability through:

• *Policy needs*: reform and adjustment of the policy framework which provides the enabling environment for vector control;

• *Institution building needs*: the strengthening of existing institutions and the arrangements between them that aim at facilitating vector control;

• *Managerial development needs*: the establishment of clear criteria and decision-making procedures to manage the vector control programme;

• *Technical strengthening*: development of the technical facilities to support vector control programmes;

• *Human-resource development needs*: the formation and in-service training of personnel in the relevant disciplines and skills.

• *Community participation*; this will ensure the sustainability of control approaches.

Needs assessments of vector control capability must identify approaches that will assure that the elements from Table 1 are properly and efficiently integrated into routine efforts of African malaria control programmes, since the elements listed in the table inherently encompass policy, institution building, managerial development, technical strengthening, human resources, and community participation needs.

### Achieving momentum for the IVM movement

IVM in the Africa region was launched at a regional workshop of policy makers and vector control specialists in Harare in February 2001. From that workshop a strategic plan was further developed to initiate the implementation of IVM in Africa.

Subsequently, a series of regional IVM training workshops were organized in Nairobi, Kenya in 2002 and 2004, and in Accra, Ghana in 2003. A total of 31 participants from 23 countries, including entomologists and vector control specialists working with Ministry of Health disease control programmes and Ministries of the Environment, attended the workshops [61].

National plans of action were prepared and implementation of IVM was started in 16 countries. Vector-control needs assessments and the organization of national consensus workshops on IVM have been conducted in nine countries. In order to support all these efforts, the "Partnership for IVM in Africa", called "IVM Africa," was founded in Harare with six organizations initially: WHO, UNEP, ICIPE, the USAID Environmental Health Project (EHP), The Hashimoto Initiative, and the Panel of Experts on Environmental Management (PEEM) [62].

There is a rapidly increasing number of programmes incorporating ITNs with IRS [63]. For example, in Boiko Island in Equatorial Guinea, ITNs have been used for over 13 years and, in 2004, an IRS campaign was also initiated [64]. Additionally, through the U.S. Government President's Malaria Initiative, vector control operations are being supported in 15 African countries within the framework of IVM, including the distribution and promotion of ITNs generally and IRS in targeted communities (6 million LLINs and 17 million persons covered by IRS by end of 2007). As well, in some specific situations larval source management is being used in combination with ITNs and IRS (e.g. Dar es Salaam). Capacity for entomological monitoring is a key feature of the IVM framework. This has lagged behind the scale-up of IRS and ITNs, but includes collaboration with NMCPs and national research institutions to conduct vector density and insecticide resistance monitoring, and in some countries support for bio-assays to judge the duration of effectiveness of IRS application on different wall surfaces.

The Global Malaria Business Plan and a recent technical review of Global Malaria Control and Elimination [28] recognize that as programmes scale up from providing ITNs to vulnerable individuals to community-wide interventions, the focus shifts from personal protection to vector control requiring not only a large financial investment in commodities and deployment, but an investment in human resources for planning, targeting, monitoring and evaluating the various control interventions. Furthermore, "Sustained Control" of 15–30 years implies the need for a new generation of public health professionals with technical skills in malaria control. Now is the time to strengthen and provide links between the national academic and research institutions and the NMCPs, both for academic and technical training.

### Recommendations for the future of IVM for malaria control in Africa

There is a need to show that IVM approaches are being implemented and that they are making an impact. In addition to the basic requirements for implementing IVM at the national level for malaria control, further coordinated national and international efforts and financial support are needed to achieve the following:

• Strengthen capacity-building for IVM at national level, improve the scope and quality of regional IVM training initiatives, and support post-graduate education.

• Further promote interdisciplinary integration and inter-sectoral cooperation by engaging appropriate stake-holders at the national level, including community groups and NGOs, and engaging experts from outside traditional entomological and public health frameworks for vector-borne disease control.

• Promote the incorporation of emerging technologies in database management, information systems, and communication into the IVM strategy for better national and regional level operations.

• Develop novel training models that also have interaction components among entomologists in the field, health-care workers in the clinics and decision-makers in national and local government offices.

• Support research that further elaborates approaches and tactics needed to combat malaria, especially for low-transmission settings where national programmes have successfully reduced levels of transmission and are now pursuing or moving toward country-wide elimination.

• Support research in options for improving strategic management and decision-making to more comprehensively integrate IVM strategies into national malaria control programmes.

• Support international research to develop new tools for malaria vector control, including new generation insecticides, new biological control strategies, new trapping methods, and innovative methods that target mosquitoes at different points in their life history cycles (i.e., sugar-feeding, mating, oviposition, blood-feeding).

• Identify and support activities that establish long-term partnerships between national malaria control programmes and international experts and institutions to strengthen capacity building and operational research components of IVM programmes.

• Establish and fund IVM demonstration projects across different malaria-endemic environments to illustrate the importance of integrated IVM approaches for vector-borne disease control.

• Increase research on the financial and economic costs of IVM including projected estimates of IVM, real-world cost-effectiveness calculations of IVM programmes from demonstration projects, and financial and economic models related to scaling-up IVM in Africa.

## List of abbreviations used

IVM: Integrated Vector Management; ITNs: Insecticide-treated bednets; IRS: Indoor Residual spraying; WHO: World Health Organization; CDC: Centers for Disease Control; FAO: Food and Agriculture Organization; NGOs: Non-governmental organizations; UNEP: United Nations Environment Programme; ICIPE: International Centre of Insect Physiology and Ecology; USAID: United States Agency for International Development; LLINs: Long-lasting insecticide-treated bednets; NMCPs: National Malaria Control Programmes.

## Competing interests

The authors declare that they have no competing interests.

## Authors' contributions

JCB conceived the review concept, organized the draft sections, co-wrote sections of the draft and edited the overall manuscript. JK participated in the conception, helped organize the draft sections, co-wrote sections of the draft and helped in editing the overall manuscript. JIG participated in the conception and helped in editing the overall manuscript. MBM participated in the conception, co-wrote sections of the draft, and helped in editing the overall manuscript. DEI co-wrote and edited sections of the manuscript and formatted the draft for submission. RJN participated in the conception, provided specific IVM information, co-wrote sections of the manuscript and helped in editing the overall manuscript.
